# The role of task shifting in reforming the working styles of pediatricians in Japan: A questionnaire survey

**DOI:** 10.1097/MD.0000000000030167

**Published:** 2022-09-02

**Authors:** Masatoshi Ishikawa

**Affiliations:** a Faculty of Medicine, University of Tsukuba, Tsukuba, Ibaraki, Japan.

## Abstract

We aimed to shorten the working hours of pediatricians who are regularly experiencing overwork in Japan, recommended tasks for task shifting must be identified, and the impact of promoting task shifting on both the quality of medical care and working hours must be examined. Characteristics of the pediatric department must also be considered. A questionnaire survey was conducted with pediatricians working in hospitals across Japan. A multiple logistic regression analysis was performed with pediatricians who did not recommend task shifting in the workplace as the explained variable and the attributes of the responding pediatricians (gender, age, primary workplace, number of pediatricians, pediatric medical management fee) as the explanatory variables. Details about the tasks recommended for task shifting and the impact of recommending task shifting on the quality of medical care and working hours were described. Questionnaires were sent nationwide to 848 hospitals that calculated pediatric inpatient medical management fees and received responses from 1539 pediatricians in 416 hospitals (response rate: 49%). As a characteristic of 231 (15%) doctors who thought that the task shift had not progressed at all at their place of employment, significant positive associations were found in men, working at national and public university hospitals, private university hospitals, and private hospitals and pediatric inpatient medical care management fee 1 hospitals. Task shifting was not recommended overall, as the task items that a majority of pediatricians marked as “transferred” were limited to “medication instructions” and “intravenous injection of antibiotics, etc” More than half of the respondents (60%, a total of 921 doctors) reported that the quality of medical care improved slightly or significantly when task shifting was promoted. The most frequent response to survey items querying the number of work hours that could be shortened through task shifting was “1 to 2 hours.” The tasks suitable for task shifting were identified based on the characteristics of participants’ pediatric departments. Results suggest that task shifting was not recommended in university hospitals and that promotion of task shifting could improve the quality of medical care and reduce the working hours of pediatricians.

## 1. Introduction

According to the World Health Organization (WHO), task shifting presents a viable solution for improving health care coverage, efficiently using available human resources, and increasing capacity quickly while training and retention programs are expanded.^[1]^

Initiatives seeking to improve medical efficiency through task shifting are promoted primarily by low and middle-income countries. The effects of task shifting have been proven in various clinical fields, including noncommunicable diseases,^[2]^ HIV/AIDS,^[3,4]^ contraceptive distribution,^[5]^ and primary care.^[6]^ In 2012, the WHO published recommendations on task shifting in maternal and newborn healthcare.^[7]^ This extant research demonstrates that task shifting is a useful measure for utilizing limited medical resources effectively.

By international standards,^[8]^ workers in Japan work longer hours. Comparing occupations, physicians have particularly long working hours. Physicians who work more than 60 hours per week account for 42% of full-time physicians, with more than 200 working days per year. Employees working more than 60 hours per week average 14% across all occupations, which is the highest rate.^[9]^ According to a survey of physicians working in hospitals throughout Japan, emergency department physicians recorded the highest average weekly working hours at 54 hours, followed by physicians in the neurosurgery department (53.3 hours), surgery department (52.5 hours), and pediatric department (52.0 hours). These results suggest that the pediatric department has relatively long working hours.^[10]^

In 2016, the Ministry of Health, Labour and Welfare established the Study Group on Working Style Reform for Physicians; in 2018, Urgent Efforts to Reduce Working Hours for Physicians was published. The second proposal suggested thorough management of working hours, utilization of existing occupational health systems, and promotion of task shifting, all as efforts to shorten the working hours of physicians. The promotion of task shifting involved initial medical care; explanations of tests, procedures, and admissions; drug explanations and medication instructions; venous blood sampling; intravenous injections; securing of intravenous lines; urinary catheter placement; completion of medical certificates and referral forms; and transfer of patients to other professionals.^[11]^ In response, a document submitted by the Japan Pediatric Society to a panel of the Ministry of Health, Labour and Welfare stated the pros and cons of task shifting specific to pediatric departments; however, it did not confirm the intention of pediatricians who are members of the society.^[12]^ Specifically, it stated that preexamination during initial treatment, intravenous blood sampling for newborns and infants, intravenous injections, and securing of intravenous lines should not be transferred to other occupations.

In order to shorten the working hours of pediatricians who are continually overworked in Japan, recommended tasks for task shifting must be identified and the impact of promoting task shifting on the quality of medical care and working hours examined, while concurrently considering the characteristics of the pediatric department. However, there have been no previous studies on this subject.

This study conducted a questionnaire survey of pediatricians working in hospitals across Japan to identify details of the tasks for which task shifting should be promoted, gather opinions about the impact of promoting task shifting on medical quality and working hours, and consider suggestions for health policies.

## 2. Methods

### 2.1. Ethical considerations

This study was approved by the Institutional Review Board of the University of Tsukuba (No. 1497, on 31 July 2020). Written informed consent was obtained from each participant.

### 2.2. Subjects

To identify the medical institutions that employ full-time pediatricians, 848 hospitals were surveyed nationwide that calculate pediatric inpatient medical management fees.^[13]^ In October 2020, after sending a survey request form to the pediatrics managers and doctors of those 848 hospitals, valid responses were obtained from 1539 pediatricians at 416 hospitals (response rate 49%).

### 2.3. Measurement and data analysis

Using the questionnaire method, an online survey was conducted. First, the attributes of the respondents (gender, age, hospital of primary employment, number of pediatricians, pediatric inpatient medical management fee, and regional characteristics) were cataloged; attributes are described in Table 1. Respondent ages were grouped in 5 categories: <30, 30 to 39, 40 to 49, 50 to 59, and ≥60. Hospitals were grouped into 4 categories: public hospitals, national public university hospitals, private university hospitals, and private hospitals. Workplace regions were categorized based on the 2016 classification of 344 secondary medical areas (SMAs) within Japan into 3 categories based on population size and population density combinations: urban, intermediate, and rural.^[14]^

**Table 1:** Characteristics of participants.

Number of respondents	1539	
Percentage of all pediatric doctors	14.4%	
Gender		
Men	999	64.9%
Women	540	35.1%
Age (yr)		
<30	159	10.3%
30–40	530	34.4%
40–50	483	31.4%
50–60	257	16.7%
≥60	110	7.1%
Marital status		
Yes	1225	79.6%
No	314	20.4%
Presence of children		
Yes	1018	66.1%
No	521	33.9%
Subspecialty		
Nothing in particular	410	26.6%
Neonatology	206	13.4%
Neurology	166	10.8%
Allergy	162	10.5%
Cardiovascular	144	9.4%
Hematology	128	8.3%
Endocrinology	101	6.6%
Nephrology	97	6.3%
Others	125	8.1%
Annual salary (including part-time job)		
<4 million yen	176	11.4%
<4–6 million yen	189	12.3%
<6–8 million yen	263	17.1%
<8–10 million yen	297	19.3%
<10–12 million yen	168	10.9%
<12–14 million yen	166	10.8%
<14–16 million yen	131	8.5%
More than 16 million yen	149	9.7%
Main workplace		
Public	821	53.3%
National and public university	198	12.9%
Private university	173	11.2%
Private	347	22.5%
Number of pediatricians in the workplace		
1–4 people	282	18.3%
5–9 people	485	31.5%
10–14 people	212	13.8%
15–19 people	141	9.2%
20 people or more	419	27.2%
Regional classification of the workplace		
Urban city	605	39.3%
Rural city	823	53.5%
Depopulated area	111	7.2%

An online survey conducted using the questionnaire method determined recommended tasks for task shifting and the impact of its promotion. The attributes of the respondents for measuring characteristics of the pediatric department were cataloged, as described in Table 1.

**Figure 1.**
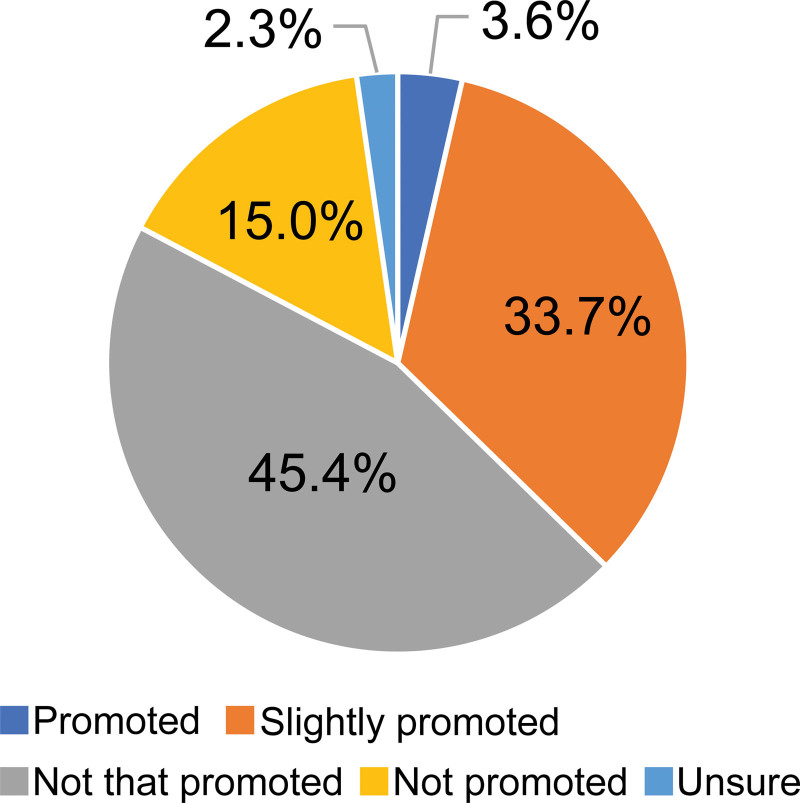
Task shifting promotion at respondent hospitals.

Next, responses to the question about respondents’ workplaces promoting task shifting (Promoted, slightly promoted, not that promoted, not promoted at all, unsure) were organized; responses are presented in Figure 1. Multivariate logistic regression analysis was performed; the physicians who answered “not promoted at all” and physician characteristics gender were explanatory variables. The characteristics of physicians who thought that task shifting was not promoted at all at the workplace were analyzed; results are presented in Table 2. The implementation status of task shifting, and the answers to questions about the pros and cons of implementing task shifting when it was not yet implemented, are described in Table 3 for each individual task. Individual task items specific to pediatrics were created referencing the Urgent Efforts to Reduce Working Hours for Physicians from the Ministry of Health, Labour and Welfare.^[15]^ Items specific to pediatrics were also created referencing the opinions of the committee of the Japan Pediatric Society.^[12]^

**Table 2:** Association between minimal task shifting and physician characteristics.

	OR	95% CI	*P* value
Gender			
Male	Reference		
Female	0.72	0.52–1.00	0.05*
Age (yr)			
<30	Reference		
30–39	1.02	0.60–1.72	0.95
40–49	1.02	0.60–1.74	0.94
50–59	0.88	0.48–1.62	0.69
>=60	1.17	0.59–2.35	0.65
Main establishment			
Public	Reference		
National and public university	2.08	1.32–3.29	<0.01*
Private university	1.84	1.07–3.17	0.03*
Private	1.60	1.10–2.31	0.01*
Pediatric inpatient medical care management fee			
Pediatric inpatient medical care management fee 1	Reference		
Pediatric inpatient medical care management fee 2	0.84	0.75–1.91	0.46
Pediatric inpatient medical care management fee 3	1.56	0.32–1.28	0.21
Pediatric inpatient medical care management fee 4	1.20	0.49–1.39	0.47
Pediatric inpatient medical care management fee 5	0.41	1.26–4.73	0.01*
Workplace			
Urban	Reference		
Intermediate	0.79	0.58–1.09	0.15
Rural	0.53	0.25–1.15	0.11

The characteristics of physicians who thought that task shifting was not promoted at all at the workplace were analyzed; results are presented in Table 2. The analysis concluded that men (control group: women), national and public university hospitals, private university hospitals, private hospitals (control group: public hospitals), and hospitals that calculate pediatric inpatient medical management fee 1 had significantly higher odds ratios.

OR = odds ratio; CI = confidence interval.

* *P* < .05.

**Table 3:** Implementation status and future direction of task shifting (%).

						
1. Substitute input	Transferred	To be mostly transferred	To be partially transferred	Not to be transferred	Neither	Total
Initial interview (initial consultation)	20%	26%	34%	17%	4%	100%
Order of test, prescription, treatment, etc	5%	24%	42%	26%	3%	100%
Reservation for hospitalization and surgery	8%	48%	28%	11%	5%	100%
Creation of medical certificate and referral form	12%	33%	34%	18%	3%	100%
Creation of discharge summary	4%	32%	34%	26%	3%	100%
Filling out electronic medical record	2%	22%	44%	28%	4%	100%
Filling out school life management table	3%	40%	33%	18%	5%	100%
Case enrollment (cancer enrollment, etc)	7%	54%	20%	9%	10%	100%
2. Explanation and instruction to patients and patient transfer						
	Transferred	To be mostly transferred	To be partially transferred	Not to be transferred	Neither	Total
Responding to telephone inquiries from patients and their families	20%	30%	34%	13%	4%	100%
Request for medical care for children	3%	24%	40%	23%	11%	100%
Explanation of examination and treatment procedure	6%	34%	37%	20%	3%	100%
Patient transfer (from laboratory to ward)	41%	35%	16%	5%	3%	100%
Patient transfer (transportation between hospitals by ambulance)	5%	23%	39%	27%	6%	100%
Meal order and nutritional guidance	29%	46%	16%	6%	3%	100%
Medication instructions	52%	32%	9%	4%	3%	100%
3. Procedures						
	Transferred	To be mostly transferred	T + E26o be partially transferred	Not to be transferred	Neither	Total
Venous blood sampling (newborn/infant)	7%	19%	43%	28%	3%	100%
Venous blood sampling (non-newborn/infant)	24%	35%	28%	11%	2%	100%
Securing venous route (newborn/infant)	5%	17%	43%	32%	3%	100%
Securing venous route (non-newborn/infant)	17%	35%	33%	12%	2%	100%
Securing contrast agent line	13%	32%	34%	18%	3%	100%
Intravenous injection of antibiotics, etc	51%	27%	14%	6%	1%	100%
Vaccine inoculation (BCG)	3%	30%	29%	33%	5%	100%
Vaccine inoculation (subdermal)	6%	37%	29%	24%	4%	100%
Vaccine inoculation (intramuscular)	4%	34%	30%	27%	4%	100%
Vaccine inoculation (oral)	16%	44%	23%	14%	3%	100%
Sample collection (stool, pharynx, posterior nasal cavity, sputum, urine pack)	32%	40%	20%	6%	2%	100%
Sample collection (blood culture)	12%	29%	32%	23%	3%	100%
Sample collection (newborn mass screening)	27%	39%	19%	9%	5%	100%
Gastric fistula replacement	4%	30%	36%	22%	9%	100%
Tracheal cannula replacement	3%	26%	38%	26%	8%	100%
Urinary catheterization, indwelling bladder catheter replacement	6%	33%	36%	18%	7%	100%
Ventilator configuration change (NICU)	1%	15%	41%	35%	7%	100%

Table 3 shows the implementation status of task shifting related to individual tasks and the pros and cons of implementing tasks when not yet implemented. The only items that the majority of pediatricians marked as “transferred” were “medication instructions” and “intravenous injection of antibiotics, and so on.”

**Figure 2.**
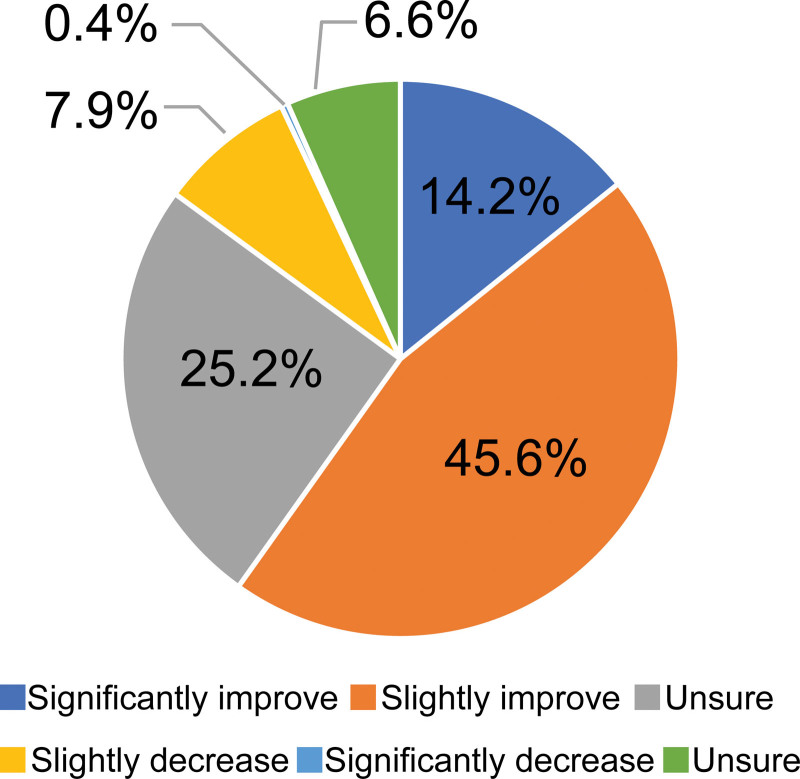
Impact on the quality of medical care if task shifting promoted.

**Figure 3.**
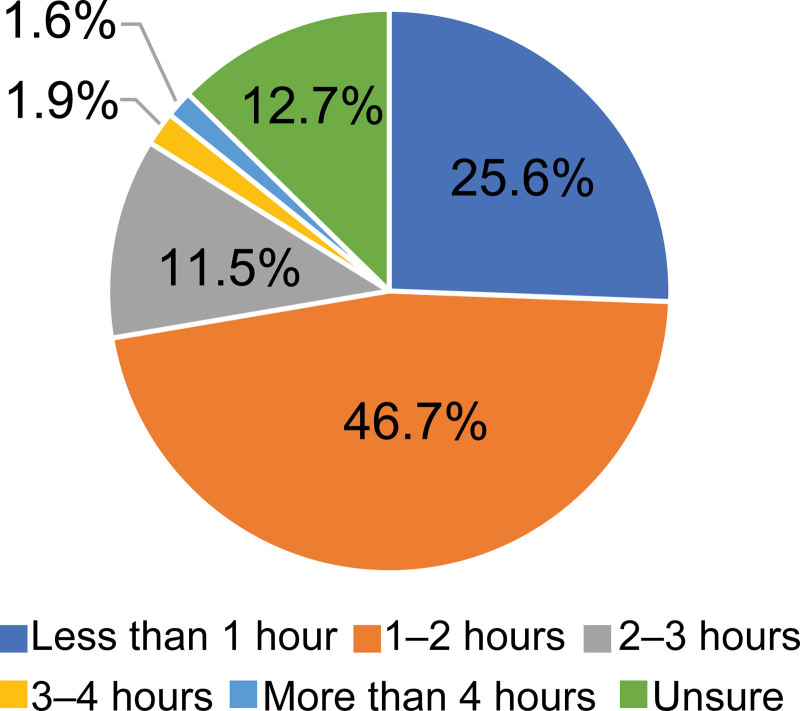
Working hours that could be shortened by task shifting.

Figure 2 was designed to present survey data related to the impact of promoting task shifting on medical quality (significantly improved, slightly improved, unchanged, slightly reduced, considerably reduced, neither). Figure 3 presents survey data related to the distribution of daily working hours that could be shortened by task shifting (<1 hour, 1 to 2 hours, 2 to 3 hours, 3 to 4 hours, more than 4 hours, unsure).

### 2.4. Statistics

STATA 15.1 was used for all statistical analyses. P-values <0.05 were considered significant.

## 3. Results

When the survey request form was sent to 848 hospitals nationwide, valid responses were obtained from 1539 pediatricians at 416 hospitals (hospital response rate: 49.0%). The 1539 pediatricians who responded account for 14.4% of the 10,614 pediatricians working in Japanese hospitals, according to the 2018 Ministry of Health, Labour and Welfare’s Statistics of Doctors, Dentists and Pharmacists.^[16]^ Table 1 shows the gender, age, marriage status, children, subspecialty, salary, main/primary workplace, number of pediatricians, and regional characteristics of the participants. As noted in Figure 1, 45.4% of survey respondents reported that task sharing was “not that promoted,” and 15.0% reported that task sharing was “not promoted.”

Next, multivariate logistic regression analysis was performed with the physicians who answered “not promoted” as the explained variable and the characteristics of the physicians (gender, age, main/primary workplace, number of pediatricians, pediatric inpatient medical management fee, and regional characteristics) as the explanatory variable. As shown in Table 2, multivariate logistic regression analysis of the characteristics of physicians who reported that task shifting was not promoted in the workplace concluded that men (control group: women), national and public university hospitals, private university hospitals, and private hospitals (control group: public hospitals), and hospitals that calculate pediatric inpatient medical management fee 1 (control group: pediatric inpatient medical management fee 5) had significantly higher odds ratios. As the number of physicians per hospital was strongly associated with the pediatric inpatient medical management fee, the number of physicians per hospital was excluded from the explanatory variables in the analysis. In terms of regional classification, although not significant, the odds ratio was higher in urban areas than in intermediate and rural areas. No significant association was found with age.

Table 3 shows the implementation status of task shifting related to individual tasks as well as the pros and cons of implementing tasks when task shifting is not yet implemented. The items that the majority of pediatricians marked as “transferred” were “medication instructions” and “intravenous injection of antibiotics.”

More than 70% of respondents answered “transferred,” “to be mostly transferred,” or “to be partially transferred” for completion of medical certificates and referral forms. In the “initial medical care” category, more than 20% of respondents answered “transferred,” and certain task shifting was being promoted.

For items related to explanations to patients, more than 70% of respondents answered “transferred,” “to be mostly transferred,” and “to be partially transferred” for all items except “request medical care for children” and “transfer of patients (transfer between hospitals by ambulance).” For the items “movement of patients (movement from laboratory to hospital ward),” “meal order and nutritional guidance,” and “medication instructions,” more than 20% of respondents answered “transferred.”

For items related to pediatric procedures, more than 70% of respondents answered “transferred,” “to be mostly transferred,” and “to be partially transferred” for all items except for “venous blood sampling (newborn/infant),” “securing intravenous route (newborn/infant),” “vaccine inoculation (BCG),” “vaccine inoculation (intramuscular injection),” “changing tracheal cannula,” and “neonatal intensive care unit (NICU).” For the items “venous blood sampling (except for newborn/infant),” “intravenous injection of antibiotics, etc,” “sample collection (stool, pharynx, posterior nasal cavity, sputum, urine pack),” and “sample collection (newborn mass screening),” more than 20% of physicians queried answered “transferred.” However, more than 30% of respondents answered “should not be transferred” for “venous blood sampling (newborn/infant),” “vaccine inoculation (BCG),” and “neonatal intensive care unit (NICU).”

As shown in Figure 2, more than half (59.8%) of physicians responded that the effect on the quality of medical care of promoting task shifting would cause slight or significant improvement. The most common answer regarding the number of working hours that could be shortened through task shifting was “1 to 2 hours” (46.7%).

## 4. Discussion

A questionnaire survey was conducted on task shifting with pediatricians working in Japanese hospitals nationwide. The results revealed that 37.3% of the responding physicians answered that task shifting was “promoted” or “slightly promoted” while 15.0% reported that task shifting was “not promoted.” It is believed that this discrepancy among physicians may be due to differences in the promotion status of work style reforms for physicians or due to differences in the implementation of task shifting of pediatric medical care tasks.

Physicians in Japanese university hospitals work in a challenging environment; many of these physicians are overworked because they are engaged in education, clinical practice, and research, while some even work unpaid.^[17,18]^ This study suggests that task shifting may not have progressed at national and public universities and private university hospitals. Young unpaid physicians providing cheap labor,^[19]^ may impede the progress of task shifting. However, optimization of labor management and promotion of task shifting at university hospitals are planned in the “Reform of Working Styles for Physicians” promoted by the Ministry of Health, Labour and Welfare. The working environment of physicians employed at Japanese university hospitals is expected to improve in the future.^[11]^

The pediatric inpatient medical management fee is ranked according to the degree of improvement within the medical care system; pediatric inpatient medical management fee 1 represents the most improved. Pediatric inpatient medical management fee 1 requires 20 or more full-time pediatricians, a large number of pediatric emergency hospitalizations, and surgeries for patients under the age of 6.^[13]^ As there are so many pediatricians, there is little promotion of task shifting to medical staff members who are not physicians.

In terms of regional differences, the more rural the area, the more likely it is that the region has fewer physicians.^[20]^ It is possible that the promotion of task shifting has contributed to alleviating the shortage of doctors.

Results on the implementation status and future direction of task shifting explain clearly that task shifting has not been recommended highly for most individual tasks, as the items that a majority of pediatricians marked as “transferred” were limited to “medication instructions” and “intravenous injection of antibiotics, etc” “Substitute input” and “explanation to patients and general procedures” were included as targets for task shifting promotion in the Urgent Efforts to Reduce Working Hours for Physicians published by the Ministry of Health, Labour and Welfare in 2018. It is expected that task shifting will progress in the future by government-led promotion the reform of working styles for physicians.^[11]^

For the items “initial interview” and “explanation of test procedures,” more than 70% of responding pediatricians responded “to be transferred” although the items are “not to be transferred” according to the Japan Pediatric Society.^[12]^ Previous studies of pediatricians have concluded that the implementation of task sharing provides new options for supporting pediatricians and creating better access to outpatient pediatric care in rural areas.^[21]^ Although individual circumstances must be considered, there may be room for a more in-depth task shifting beyond the expectations of the Japanese Pediatric Society.

This study suggests that the promotion of task shifting could improve the quality of medical care and reduce the working hours of pediatricians. When pediatricians promote task shifting, 60% of physicians said that the quality improved; only 8% said that quality decreased. Degradation of healthcare quality due to task shifting should be avoided, but it has become clear that many pediatricians who responded to this survey do not believe that degradation occurs with task shifting.

The highest number of physician respondents (approximately 47%) answered “1 to 2 hours” followed by responses of “less than 1 hour” (approximately 26%) when asked about the number of daily working hours that could be shortened through task shifting. Pediatricians work for 52 hours per week, which is relatively longer than physicians in other departments.^[10]^ With this fact and survey results in mind, if task shifting can shorten daily working hours by 2 hours, total working hours will improve and be closer to the legal working hours of 40 hours per week. According to a report from the Health and Global Policy Institute in Japan, due to the longer work hours for pediatricians in Japan, fewer doctors choose pediatrics.^[22]^ Thus, it is necessary to promote work style reforms for pediatricians and eliminate the uneven distribution of doctors.

There are several limitations to this study. First, the response rate was 49.0% based on the number of hospitals in Japan and 14.4% based on the number of physicians in Japan. Therefore, study results may not necessarily reflect the nationwide intentions of pediatricians. Second, a survey was conducted among pediatricians; however, to promote task shifting, the ideas of nurses and clerical workers who will perform task shifting and experience the challenges in terms of regulations and skills, should also be solicited. Japan follows a nurse practitioner system similar to the United States. The current findings propagate policy considerations for task shifting given that appropriate training is provided to nurse practitioners regarding specialized tasks that should be transferred.^[23]^ However, such an examination has yet to be conducted.

Reform of the working style of physicians is a global issue.^[7]^ To promote task shifting, more specific policies about medical systems and education are needed.
